# 2,5-Dimethoxy­benzaldehyde thio­semicarbazone

**DOI:** 10.1107/S1600536808035198

**Published:** 2008-11-08

**Authors:** Hoong-Kun Fun, Samuel Robinson Jebas, E. Deepak D’Silva, P. S. Patil, S. M. Dharmaprakash

**Affiliations:** a X-ray Crystallography Unit, School of Physics, Universiti Sains Malaysia, 11800 USM, Penang, Malaysia; b Department of Studies in Physics, Mangalore University, Mangalagangotri, Mangalore 574 199, India

## Abstract

In the title mol­ecule, C_10_H_13_N_3_O_2_S, the dihedral angle between benzene and –N—C(=S)—N—N=C– planes is 9.20 (6)°. The two meth­oxy groups are coplanar with the benzene ring [C—O—C—C torsion angles of −2.31 (18) and −6.45 (17)°]. In the crystal structure, mol­ecules are linked by inter­molecular N—H⋯S, N—H⋯O and C—H⋯O hydrogen bonds, forming a three-dimensional network.

## Related literature

For the biomedical properties of thio­semicarbazones, see: Beraldo & Gambino (2004 ▶). For bond-length data, see: Allen *et al.* (1987 ▶).
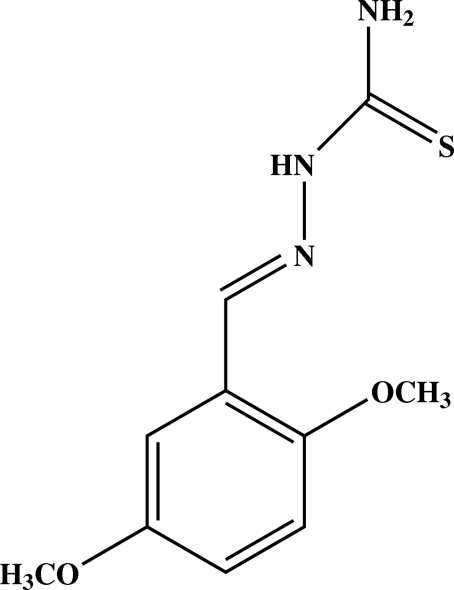


## Experimental

### 

#### Crystal data

C_10_H_13_N_3_O_2_S
*M* *_r_* = 239.29
Orthorhombic, 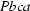

*a* = 11.0713 (1) Å
*b* = 13.0603 (2) Å
*c* = 15.7808 (2) Å
*V* = 2281.82 (5) Å^3^
*Z* = 8
Mo *K*α radiation
μ = 0.27 mm^−1^
*T* = 100.0 (1) K
0.34 × 0.28 × 0.22 mm

#### Data collection

Bruker SMART APEXII CCD area-detector diffractometer
Absorption correction: multi-scan (*SADABS*; Bruker, 2005 ▶) *T*_min_ = 0.912, *T*_max_ = 0.943
18486 measured reflections
3486 independent reflections
2834 reflections with *I* > 2σ(*I*)
*R*_int_ = 0.043

#### Refinement

*R*[*F* ^2^ > 2σ(*F* ^2^)] = 0.037
*wR*(*F* ^2^) = 0.097
*S* = 1.06
3486 reflections
157 parameters
2 restraints
H atoms treated by a mixture of independent and constrained refinement
Δρ_max_ = 0.40 e Å^−3^
Δρ_min_ = −0.27 e Å^−3^

### 

Data collection: *APEX2* (Bruker, 2005 ▶); cell refinement: *APEX2* and *SAINT* (Bruker, 2005 ▶); data reduction: *SAINT*; program(s) used to solve structure: *SHELXTL* (Sheldrick, 2008 ▶); program(s) used to refine structure: *SHELXTL*; molecular graphics: *SHELXTL*; software used to prepare material for publication: *SHELXTL* and *PLATON* (Spek, 2003 ▶).

## Supplementary Material

Crystal structure: contains datablocks global, I. DOI: 10.1107/S1600536808035198/ci2701sup1.cif

Structure factors: contains datablocks I. DOI: 10.1107/S1600536808035198/ci2701Isup2.hkl

Additional supplementary materials:  crystallographic information; 3D view; checkCIF report

## Figures and Tables

**Table 1:** Hydrogen-bond geometry (Å, °)

*D*—H⋯*A*	*D*—H	H⋯*A*	*D*⋯*A*	*D*—H⋯*A*
N2—H1*N*2⋯S1^i^	0.84 (4)	2.718 (18)	3.5375 (12)	166 (2)
N3—H2*N*3⋯S1^ii^	0.86 (1)	2.811 (13)	3.5047 (12)	139 (1)
N3—H1*N*3⋯O2^iii^	0.86 (1)	2.145 (11)	2.9617 (15)	159 (2)
C3—H3*A*⋯O1^iv^	0.93	2.51	3.3027 (16)	143

Symmetry codes: (i) 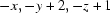; (ii) 
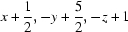
; (iii) 
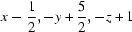
; (iv) 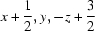.

**Fig. 1.**
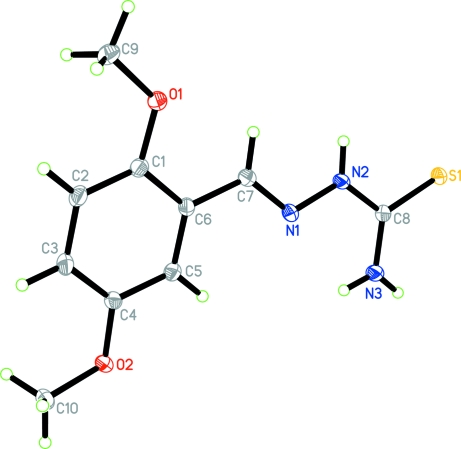
The molecular structure of the title compound, showing 50% probability displacement ellipsoids and the atom-numbering scheme.

**Fig. 2.**
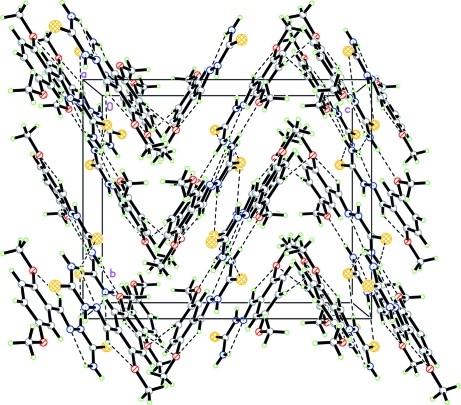
The crystal packing of the title compound, viewed along the a axis. Dashed lines indicate hydrogen bonds.

## supplementary crystallographic information

### Comment

Thiosemicarbazones are of great interest because of their profound biomedical properties (Beraldo *et al.*, 2004). Flexibility and bioactivity of these compounds arise due to the presence of amino group (–N═CH–) in addition to thioamino moieties present in the skeleton of the molecule. We have synthesized the title compound and its crystal structure is reported here.

The bond lengths in the title molecule (Fig.1) are found to have normal values (Allen *et al.*, 1987). The two methoxy groups are coplanar with the benzene ring, with C9—O1—C1—C2 and C10—O2—C4—C3 torsion angles of -2.31 (18) and -6.45 (17)°, respectively. The dihedral angle between the C1—C6 and S1/N1—N3/C7/C8 planes is 9.20 (6)°.

In the crystal packing, the molecules are linked together by intermolecular N—H···S, N—H···O and C—H···O hydrogen bonds (Table 1) to form a three-dimensional network (Fig.2).

### Experimental

The title compound was synthesized by refluxing 2,5-dimethoxy benzaldehyde (0.075 mol) and thiosemicarbazone (0.05 mol) in methanol (100 ml) for 2 h. The solution was then allowed to cool, poured into a beaker containing water and stirred for 30 min. The product was separated by filtration and the crude sample obtained was recrystallized twice from hot methanol.

### Refinement

N-bound H atoms were located in a difference map and were refined with an N—H distance restraint of 0.86 (1) Å. C-bound H atoms were placed in calculated positions (C—H = 0.93–0.96 Å) and refined using a riding model with *U*_iso_(H) = 1.2–1.5*U*_eq_(C).

### Crystal data

**Table:**

C_10_H_13_N_3_O_2_S	*F*_000_ = 1008
*M**_r_* = 239.29	*D*_x_ = 1.393 Mg m^−^^3^
Orthorhombic, *P**b**c**a*	Mo *K*α radiation λ = 0.71073 Å
Hall symbol: -P 2ac 2ab	Cell parameters from 4233 reflections
*a* = 11.0713 (1) Å	θ = 2.6–30.0º
*b* = 13.0603 (2) Å	µ = 0.27 mm^−^^1^
*c* = 15.7808 (2) Å	*T* = 100.0 (1) K
*V* = 2281.82 (5) Å^3^	Block, colourless
*Z* = 8	0.34 × 0.28 × 0.22 mm

### Data collection

**Table:**

Bruker SMART APEXII CCD area-detector diffractometer	3486 independent reflections
Radiation source: fine-focus sealed tube	2834 reflections with *I* > 2σ(*I*)
Monochromator: graphite	*R*_int_ = 0.043
*T* = 100.0(1) K	θ_max_ = 30.5º
φ and ω scans	θ_min_ = 2.6º
Absorption correction: multi-scan(SADABS; Bruker, 2005)	*h* = −15→11
*T*_min_ = 0.912, *T*_max_ = 0.943	*k* = −14→18
18486 measured reflections	*l* = −22→19

### Refinement

**Table:**

Refinement on *F*^2^	Secondary atom site location: difference Fourier map
Least-squares matrix: full	Hydrogen site location: inferred from neighbouring sites
*R*[*F*^2^ > 2σ(*F*^2^)] = 0.037	H atoms treated by a mixture of independent and constrained refinement
*wR*(*F*^2^) = 0.097	*w* = 1/[σ^2^(*F*_o_^2^) + (0.0435*P*)^2^ + 0.8407*P*] where *P* = (*F*_o_^2^ + 2*F*_c_^2^)/3
*S* = 1.06	(Δ/σ)_max_ = 0.002
3486 reflections	Δρ_max_ = 0.40 e Å^−^^3^
157 parameters	Δρ_min_ = −0.27 e Å^−^^3^
2 restraints	Extinction correction: none
Primary atom site location: structure-invariant direct methods	

### Special details

**Table:**

Experimental. The data was collected with the Oxford Cyrosystem Cobra low-temperature attachment.
Geometry. All e.s.d.'s (except the e.s.d. in the dihedral angle between two l.s. planes) are estimated using the full covariance matrix. The cell e.s.d.'s are taken into account individually in the estimation of e.s.d.'s in distances, angles and torsion angles; correlations between e.s.d.'s in cell parameters are only used when they are defined by crystal symmetry. An approximate (isotropic) treatment of cell e.s.d.'s is used for estimating e.s.d.'s involving l.s. planes.
Refinement. Refinement of *F*^2^ against ALL reflections. The weighted *R*-factor *wR* and goodness of fit *S* are based on *F*^2^, conventional *R*-factors *R* are based on *F*, with *F* set to zero for negative *F*^2^. The threshold expression of *F*^2^ > σ(*F*^2^) is used only for calculating *R*-factors(gt) *etc*. and is not relevant to the choice of reflections for refinement. *R*-factors based on *F*^2^ are statistically about twice as large as those based on *F*, and *R*- factors based on ALL data will be even larger.

### Fractional atomic coordinates and isotropic or equivalent isotropic displacement parameters (Å^2^)

**Table:**

	*x*	*y*	*z*	*U*_iso_*/*U*_eq_	
S1	−0.01083 (3)	1.16558 (3)	0.45083 (2)	0.01697 (9)	
O1	0.35907 (8)	0.80559 (7)	0.68704 (6)	0.0191 (2)	
O2	0.72695 (8)	1.07776 (7)	0.61508 (6)	0.0165 (2)	
N1	0.28066 (10)	1.06262 (9)	0.56220 (6)	0.0150 (2)	
N2	0.16335 (10)	1.07175 (9)	0.53426 (7)	0.0157 (2)	
N3	0.21269 (11)	1.23005 (9)	0.48405 (7)	0.0178 (2)	
C1	0.45590 (12)	0.86889 (10)	0.67348 (7)	0.0150 (2)	
C2	0.57116 (12)	0.85165 (10)	0.70424 (8)	0.0174 (3)	
H2A	0.5868	0.7939	0.7370	0.021*	
C3	0.66395 (12)	0.92047 (10)	0.68638 (8)	0.0175 (3)	
H3A	0.7414	0.9085	0.7070	0.021*	
C4	0.64053 (11)	1.00689 (10)	0.63780 (7)	0.0144 (2)	
C5	0.52450 (12)	1.02595 (10)	0.60844 (7)	0.0145 (2)	
H5A	0.5091	1.0846	0.5768	0.017*	
C6	0.43114 (11)	0.95785 (10)	0.62609 (7)	0.0138 (2)	
C7	0.30819 (12)	0.97659 (10)	0.59612 (8)	0.0153 (2)	
H7A	0.2497	0.9259	0.6017	0.018*	
C8	0.13017 (11)	1.15736 (10)	0.49192 (7)	0.0142 (2)	
C9	0.37938 (13)	0.71375 (11)	0.73357 (9)	0.0231 (3)	
H9A	0.3049	0.6766	0.7387	0.035*	
H9B	0.4092	0.7304	0.7890	0.035*	
H9C	0.4377	0.6724	0.7044	0.035*	
C10	0.84490 (12)	1.06643 (11)	0.65093 (8)	0.0184 (3)	
H10A	0.8965	1.1200	0.6303	0.028*	
H10B	0.8777	1.0012	0.6350	0.028*	
H10C	0.8397	1.0705	0.7116	0.028*	
H1N2	0.1156 (16)	1.0222 (14)	0.5376 (11)	0.024 (4)*	
H2N3	0.2833 (10)	1.2200 (13)	0.5055 (10)	0.025 (4)*	
H1N3	0.1967 (18)	1.2835 (10)	0.4545 (10)	0.036 (5)*	

### Atomic displacement parameters (Å^2^)

**Table:**

	*U*^11^	*U*^22^	*U*^33^	*U*^12^	*U*^13^	*U*^23^
S1	0.01117 (16)	0.01533 (17)	0.02441 (16)	0.00118 (11)	−0.00228 (12)	0.00302 (11)
O1	0.0150 (5)	0.0161 (5)	0.0261 (5)	0.0003 (4)	0.0023 (4)	0.0075 (4)
O2	0.0115 (4)	0.0155 (5)	0.0224 (4)	−0.0017 (3)	−0.0022 (3)	0.0021 (3)
N1	0.0109 (5)	0.0158 (5)	0.0184 (5)	0.0003 (4)	−0.0023 (4)	0.0008 (4)
N2	0.0107 (5)	0.0134 (5)	0.0231 (5)	−0.0015 (4)	−0.0029 (4)	0.0036 (4)
N3	0.0139 (5)	0.0143 (5)	0.0251 (5)	−0.0012 (4)	−0.0037 (4)	0.0035 (4)
C1	0.0154 (6)	0.0134 (6)	0.0161 (5)	0.0008 (5)	0.0028 (5)	0.0010 (4)
C2	0.0187 (7)	0.0140 (6)	0.0196 (5)	0.0031 (5)	−0.0001 (5)	0.0035 (4)
C3	0.0143 (6)	0.0176 (6)	0.0205 (6)	0.0035 (5)	−0.0029 (5)	0.0008 (5)
C4	0.0129 (6)	0.0141 (6)	0.0161 (5)	0.0002 (5)	0.0007 (4)	−0.0015 (4)
C5	0.0152 (6)	0.0131 (6)	0.0154 (5)	0.0010 (5)	−0.0009 (4)	0.0013 (4)
C6	0.0125 (6)	0.0137 (6)	0.0151 (5)	0.0016 (5)	0.0000 (4)	0.0004 (4)
C7	0.0130 (6)	0.0152 (6)	0.0176 (5)	−0.0009 (5)	0.0001 (4)	0.0013 (4)
C8	0.0132 (6)	0.0137 (6)	0.0156 (5)	0.0010 (4)	0.0009 (4)	−0.0008 (4)
C9	0.0223 (7)	0.0168 (7)	0.0301 (7)	0.0002 (5)	0.0039 (6)	0.0085 (5)
C10	0.0127 (6)	0.0206 (7)	0.0218 (6)	0.0005 (5)	−0.0025 (5)	−0.0017 (5)

### Geometric parameters (Å, °)

**Table:**

S1—C8	1.6938 (13)	C2—H2A	0.93
O1—C1	1.3706 (16)	C3—C4	1.3888 (18)
O1—C9	1.4242 (16)	C3—H3A	0.93
O2—C4	1.3788 (15)	C4—C5	1.3881 (17)
O2—C10	1.4308 (15)	C5—C6	1.3917 (18)
N1—C7	1.2813 (16)	C5—H5A	0.93
N1—N2	1.3768 (15)	C6—C7	1.4617 (18)
N2—C8	1.3533 (16)	C7—H7A	0.93
N2—H1N2	0.838 (18)	C9—H9A	0.96
N3—C8	1.3235 (17)	C9—H9B	0.96
N3—H2N3	0.861 (9)	C9—H9C	0.96
N3—H1N3	0.857 (9)	C10—H10A	0.96
C1—C2	1.3837 (19)	C10—H10B	0.96
C1—C6	1.4087 (17)	C10—H10C	0.96
C2—C3	1.3938 (19)		
			
C1—O1—C9	117.71 (10)	C6—C5—H5A	119.8
C4—O2—C10	117.46 (10)	C5—C6—C1	119.26 (12)
C7—N1—N2	115.73 (11)	C5—C6—C7	121.33 (11)
C8—N2—N1	119.06 (11)	C1—C6—C7	119.41 (12)
C8—N2—H1N2	119.9 (12)	N1—C7—C6	120.23 (12)
N1—N2—H1N2	120.5 (12)	N1—C7—H7A	119.9
C8—N3—H2N3	118.7 (11)	C6—C7—H7A	119.9
C8—N3—H1N3	119.5 (14)	N3—C8—N2	116.85 (12)
H2N3—N3—H1N3	121.6 (17)	N3—C8—S1	123.70 (10)
O1—C1—C2	124.64 (11)	N2—C8—S1	119.44 (10)
O1—C1—C6	115.35 (11)	O1—C9—H9A	109.5
C2—C1—C6	120.00 (12)	O1—C9—H9B	109.5
C1—C2—C3	120.25 (12)	H9A—C9—H9B	109.5
C1—C2—H2A	119.9	O1—C9—H9C	109.5
C3—C2—H2A	119.9	H9A—C9—H9C	109.5
C4—C3—C2	119.87 (12)	H9B—C9—H9C	109.5
C4—C3—H3A	120.1	O2—C10—H10A	109.5
C2—C3—H3A	120.1	O2—C10—H10B	109.5
O2—C4—C5	115.78 (11)	H10A—C10—H10B	109.5
O2—C4—C3	124.03 (11)	O2—C10—H10C	109.5
C5—C4—C3	120.19 (12)	H10A—C10—H10C	109.5
C4—C5—C6	120.39 (12)	H10B—C10—H10C	109.5
C4—C5—H5A	119.8		
			
C7—N1—N2—C8	−174.96 (11)	C4—C5—C6—C1	−0.54 (18)
C9—O1—C1—C2	−2.31 (18)	C4—C5—C6—C7	179.74 (11)
C9—O1—C1—C6	179.11 (11)	O1—C1—C6—C5	−179.27 (11)
O1—C1—C2—C3	179.55 (12)	C2—C1—C6—C5	2.07 (18)
C6—C1—C2—C3	−1.93 (19)	O1—C1—C6—C7	0.45 (17)
C1—C2—C3—C4	0.25 (19)	C2—C1—C6—C7	−178.20 (11)
C10—O2—C4—C5	173.96 (11)	N2—N1—C7—C6	178.52 (10)
C10—O2—C4—C3	−6.45 (17)	C5—C6—C7—N1	−8.95 (18)
C2—C3—C4—O2	−178.28 (11)	C1—C6—C7—N1	171.33 (11)
C2—C3—C4—C5	1.30 (19)	N1—N2—C8—N3	−3.50 (17)
O2—C4—C5—C6	178.47 (11)	N1—N2—C8—S1	175.42 (9)
C3—C4—C5—C6	−1.14 (19)		

### Hydrogen-bond geometry (Å, °)

**Table:**

*D*—H···*A*	*D*—H	H···*A*	*D*···*A*	*D*—H···*A*
N2—H1N2···S1^i^	0.84 (4)	2.718 (18)	3.5375 (12)	166 (2)
N3—H2N3···S1^ii^	0.86 (1)	2.811 (13)	3.5047 (12)	139 (1)
N3—H1N3···O2^iii^	0.86 (1)	2.145 (11)	2.9617 (15)	159 (2)
C3—H3A···O1^iv^	0.93	2.51	3.3027 (16)	143

Symmetry codes: (i) −*x*, −*y*+2, −*z*+1; (ii) *x*+1/2, −*y*+5/2, −*z*+1; (iii) *x*−1/2, −*y*+5/2, −*z*+1; (iv) *x*+1/2, *y*, −*z*+3/2.
